# COVID-19 during pregnancy and adverse outcomes: Concerns and recommendations from The Brazilian Teratology Information Service

**DOI:** 10.1590/1678-4685-GMB-2020-0224

**Published:** 2021-03-10

**Authors:** Fernanda Sales Luiz Vianna, Lucas Rosa Fraga, Alberto Mantovani Abeche, André Anjos Da Silva, Maria Teresa Vieira Sanseverino, Lavinia Schuler-Faccini

**Affiliations:** 1 Universidade Federal do Rio Grande do Sul, Departamento de Genética, Programa de Pós-Graduação em Genética e Biologia Molecular, Porto Alegre, RS, Brazil.; 2 Hospital de Clínicas de Porto Alegre, Sistema Nacional de Informações sobre Agentes Teratogênicos, Porto Alegre, RS, Brazil.; 3 Hospital de Clínicas de Porto Alegre, Laboratório de Medicina Genômica, Serviço de Pesquisa Experimental, Porto Alegre, RS, Brazil.; 4 Universidade Federal do Rio Grande do Sul, Faculdade de Medicina, Programa de Pós-Graduação em Ciências Médicas, Porto Alegre, RS, Brazil.; 5 Universidade Federal do Rio Grande do Sul, Instituto de Ciências Básicas da Saúde, Departamento de Ciências Morfológicas, Porto Alegre, RS, Brazil.; 6 Universidade do Vale do Taquari (Univates), Programa de Pós Graduação em Ciências Médicas, Lajeado, RS, Brazil.

**Keywords:** Teratogenesis, birth defects, MERS, SARS, maternal complications

## Abstract

SARS-CoV-2 virus was first identified in the beginning of 2020 and has spread all over the world, causing the Coronavirus Disease 2019 (COVID-19) pandemic. The virus is a member of the Coronavirus family, which includes viruses that cause common cold, Middle East Respiratory Syndrome (MERS) and Severe Acute Respiratory Syndrome (SARS). MERS and SARS are known by causing adverse events in pregnancy. Considering that SARS-CoV-2 is a new infection agent, little is known about the risk of its infection to human embryo/fetal development. However, SARS and MERS were associated with negative outcomes, such as miscarriage, preterm birth, intrauterine growth restriction and perinatal death. Here, we raise concerns and possibilities related the harmful potential of SARS-CoV-2 and COVID-19 to pregnancy, discussing symptoms, immunological changes during pregnancy, SARS-CoV-2 mutation rate (and the risks related to it). Finally, we point out recommendations to be performed by the scientific community and health care workers in order to identify and to manage potential risks to pregnant women and their babies.

Since the beggining of 2020, COVID-19 (Coronavirus Disease 2019) has become the main concern worldwide, once its pandemic has reached all continents, infecting and causing death of thousands of people. Moreover, COVID-19 is causing different social, economic and psychological impact. Especially from the medical point of view, COVID-19 is a huge challenge. Patients with this condition can be asymptomatic and the spectrum of illness ranges from mild disease to severe disease (dyspnea, hypoxia or >50% lung involvement on imaging within 24-48 hours) (Wu and McGoogan, 2020). Moreover, so far, there is no specific treatment available for COVID-19 and the current treatment depends on supportive care of the infected patients. 

The COVID-19 causative agent SARS-CoV-2 is a member of the coronavirus family (Coronaviridae), which includes other viruses that cause other infectious conditions, such as common cold (229E, NL63, OC43 and HKU1), Middle East Respiratory Syndrome (MERS) and Severe Acute Respiratory Syndrome (SARS) (Su et al., 2016; Cui *et al*., 2019). Both MERS and SARS are related to more severe respiratory symptoms and are associated mainly with nosocomial spread, whereas SARS-CoV-2 is much more widely transmitted in the community (Munster et al; 2020). Taking into account that SARS-Cov-2 is a new species of virus, much less is known about the risk of the infection in different scenarios, especially during pregnancy and possible damages to the developing baby. 

Many studies have been performed with different approaches trying to understand the impact of COVID-19 to both mother and baby. So far, most of them are reassuring and they have shown no associations between pregnancy and severity of COVID-19 in pregnant women or birth defects in newborns (Chen D et al., 2020; Chen H *et al*., 2020; Liu D et al., 2020; Li N *et al*., 2020; Wadman, 2020). However, some of them have raised concerns. One study found a higher frequency of maternal complications in positive or suspected pregnant women with COVID-19 than in the control group. None of these complications were considered severe (Liu D *et al*., 2020). A study published by The United States Centers of Disease Control (CDC) evaluated approximately 400,000 symptomatic women for COVID-19, aged 15 to 44 years, and identified that admission to intensive care units, need of intubation or extracorporeal oxygenation and even death were more frequent in pregnant women than in non-pregnant women. Even so, the absolute risks of these outcomes, including maternal death, are still less than 1% in this study (Zambrano et al., 2020). Moreover, a Brazilian study described a 12.7% mortality rate for pregnant and postpartum women with COVID-19 evaluating data from February 26, 2020 until June 18, 2020 using the Brazilian Ministry of Health’s Acute Respiratory Distress Syndrome (ARDS) Surveillance System (Takemoto et al., 2020). This study showed that ARDS caused by COVID-19 was diagnosed in 978 pregnant and postpartum women in Brazil during the study period, of which 124 deaths were in pregnant or postpartum women (i.e. 3.4 times higher than the total number of COVID-19-related maternal deaths reported worldwide) (Takemoto *et al*., 2020). 

Studies that investigated vertical transmission did not find viral presence in placenta and newborns from pregnant women with COVID-19 (Fan et al., 2020; Lamouroux et al., 2020; Lopes de Sousa et al., 2020). On the other hand, analyses of serum samples from newborns whose mothers were seropositive for SARS-CoV-2 showed that most of these newborns presented IgG antibodies against the virus, indicating a transplacental transfer of these antibodies and a neonatal protection from the disease (Flannery et al., 2020). In a systematic review assessing 755 pregnant women and 598 infants, no negative outcomes were observed, and since the studies presented low levels of evidence, the author’s conclusion was that they could not rule out a potential worsening of the clinical conditions of pregnant women infected with SARS-CoV-2 (Lopes de Sousa *et al*., 2020). However, a small retrospective study with 54 pregnant women suspected (16) or confirmed (38) with COVID-19 showed association of the disease with maternal morbidity and preterm birth. The authors have identified that 24.1% (13/54) received oxygen support, 5.5% (3/54) required invasive mechanical ventilation, and 3.8% (5/21) had deliveries - 14.3% (3/21) very preterm - indicated for severe maternal conditions related to COVID-19 disease (Sentilhes et al., 2020). Still, some systematic reviews and meta-analysis have shown a high rate of maternal and neonatal complications in pregnant women with COVID-19, including emergency cesarean, fetal distress, preterm labor and stillbirth. In addition, although some reports of the presence of viral RNA in some newborns, the risk of neonatal infection is low (Capobianco et al., 2020; Galang et al., 2020; Gao et al., 2020). From these results, it should be recommended to counsel pregnant women about these risks.

Due to the limited and very recent data, the potential for vertical transmission cannot be ruled out. The risks for pregnant women and birth defects in newborn need to be investigated, especially because there are other viral infections that cause adverse outcomes in pregnancy, even when no vertical transmission is recognized (Schwartz and Graham, 2020). Experience from other coronaviruses corroborates this hypothesis, and both SARS and MERS have been related to negative outcomes in pregnancy, such as miscarriage, preterm birth, intrauterine growth restriction and perinatal death, even without clear evidence of vertical transmission (Wong et al., 2004; Su et al., 2016; Cui et al., 2019; Di Mascio et al., 2020; Schwartz, 2020). Interestingly, data have suggested that pregnant women with COVID-19 presented less maternal and neonatal adverse events when compared to SARS and MERS. Previous reports from SARS and MERS suggest that clinical findings during pregnancy range from women with no symptoms to more severe and fatal outcomes (Shek et al., 2003; Wong *et al*., 2004; Rasmussen et al., 2020). However, it is important to mention that, for SARS, pregnancy, outcomes varied for trimester of infection (Wong *et al*., 2004), and also must be taking into account for COVID-19, mainly because most studies so far focus are on late pregnancy.

For many reasons, and not all of them yet fully understood, pregnant women in general have a higher mortality rate and complications associated with viral infections, when compared to the general population. Pneumonia due to infectious agents is an important cause of maternal morbidity and mortality, being the most prevalent non-obstetric infection in pregnancy (Racicot and Mor, 2017). Around 25% of pregnant women who develop pneumonia need to be hospitalized in intensive care units and require ventilatory support (Madinger et al., 1989). Although bacterial pneumonia is a serious disease in pregnancy, viral pneumonia has even higher levels of morbidity and mortality (Rigby and Pastorek, 1996). Physiological changes that occur during pregnancy (immunity, circulation, respiratory system) might be considered an additional concern for these women in an infection context. Physiological changes in lung function and immunity are hypothesized contribute to these more severe outcomes (Weinberger et al., 1980; Nyhan et al., 1983). Immune responses are markedly changed during pregnancy and can be affected favorably or unfavorably. Multiple sclerosis and rheumatoid arthritis, for instance, usually improve during pregnancy. However, such immunological changes can increase the severity of some viral infections. In fact, pregnancy is a challenge for the immune system. During gestation, the body needs to tolerate the development of an embryo/fetus that presents non-self antigens, and, at the same time, it is still essential to be ready for the defense of potential pathogen invasion. Thus, a successful pregnancy has to pass through immune adaptations both systemically and locally (Liu H et al., 2020). Such adaptations are not homogeneous throughout gestation, since there are different events in the different moments of pregnancy, becoming extremely dynamic and well controlled. Mor *et al*. (2017) have shown different gestational stages from the immune system point of view. In the first trimester a pro-inflammatory scenario is necessary, in order to succeed embryo implantation and placentation. During the second trimester, around the 13^th^ week of gestation, an anti-inflammatory state is established and helps the fetal growth; and at the end of pregnancy, again a pro-inflammatory state is established, preparing the body for the initiation of delivery (Mor *et al*., 2017). 

One aspect that has recently received more attention is the role of the placenta in immune regulation. Placenta and fetal membranes are selective barriers with two primary functions: nourishing and protecting the developing fetus. Recently, it has been found that the trophoblast has a significant role in regulating the immune response when there is a serious infection at the maternal-fetal interface. In addition, the placenta functions as an immunity-modulating organ that regulates the immune responses of cells present both at the implantation site and systemically (Abrahams et al., 2004; Cardenas et al., 2010; Mor and Cardenas, 2010). However, it is not yet clear what exactly the effect of viral infections is on both homeostasis at the site of implantation and the maternal systemic immune system (Silasi et al., 2015). Viral infections capable of crossing the placenta can reach the fetus and cause serious changes in its development. Viral infection of cells at the maternal-fetal interface can affect placental function, cause miscarriages, intrauterine growth restriction and preterm birth (Racicot and Mor, 2017). Viral infection of the decidua and placenta can cause the production of soluble immune factors that reach the fetus and can affect its development. Cardenas and colleagues developed an animal model to assess the consequences of a viral infection characterized by absence of transmission to the fetus. They found that maternal viral infection can lead to productive replication in the placenta and fetal inflammatory response, even when the virus is not detected in the fetus. They suggest that the viral infection of the placenta can cause a fetal inflammatory response that in turn causes organ damage and developmental deficiencies, and that the viral infection also may sensitize the pregnant mother to bacterial products and promote preterm labor (Cardenas *et al*., 2010). Another interesting finding is that placentas from third trimester COVID-19 infected mothers have shown increased features of maternal vascular malperfusion (MVM) in comparison to control. Abnormal maternal vessels and intervillous thrombi were observed, which can reflect a systemic inflammatory or hypercoagulable state influencing placental physiology (Shanes et al., 2020). A study described the pathology and clinical information on 20 placentas whose mother tested positive for the COVID-19; 10 of the 20 cases showed some evidence of fetal vascular malperfusion or fetal vascular thrombosis suggesting that maternal COVID-19 infection might be associated with propensity for thrombosis in the fetal circulation (Baergen and Heller, 2020)

From the systemic point of view, the first line of defense against coronaviruses is the recognition by the innate immune system, which activates Nuclear Factor kappa B (NF-κB) and Interferon Regulatory Factor 3 (IRF3) transcriptional activity. This leads to the expression of Type I Interferon (IFN) and pro-inflammatory cytokines (de Wit et al., 2016; Prompetchara et al., 2020). A study performed with infected human lung tissues found that, similar to SARS-CoV, SARS-CoV-2 also targeted pneumocytes (both types I and II) and alveolar macrophages. However, compared to SARS-CoV, SARS-CoV-2 did not significantly induce types I, II or III Interferons (Chu et al., 2020). In this study, SARS-CoV-2 infection did not significantly trigger any IFN response, and only activated a few proinflammatory mediators. Therefore, if SARS-CoV-2 in fact lead a low degree of innate immune activation, it could also account for the mild or even lack of symptoms in many COVID-19 patients. The authors also postulated that the suboptimally activated innate immune response would allow SARS-CoV-2 to replicate to high levels in the respiratory tract (Chu *et al*., 2020). In a scenario without an effective innate immune response, pregnant women with COVID-19 could be at a higher risk for more severe respiratory outcomes, since during pregnancy the upper respiratory tract tends to be swollen by a high level of estrogen and progesterone, and restricted lung expansion makes them susceptible to respiratory pathogens (Liu H et al., 2020). Sex hormones can also work as signaling molecules for immune responses, as long as they are within appropriate levels. Excessive estrogens and progestogens, as occurs in pregnancy, could lead to impaired lung function. On the other hand, low levels of sex hormones could be considered risk factors in men, whose steroid hormones are at lower levels, especially in older individuals, influencing an adequate inflammatory response (Mauvais-Jarvis et al., 2020). 

Pulmonary inflammation and extensive lung damage in response to infection could have repercussions to the developing embryo/fetus due to the level of the maternal inflammatory response and the levels of inflammatory cytokines. High levels of Interleukin (IL)-1, IL-6, IL-8, and tumor necrosis factor (TNF)-α could affect the development of the fetal brain and circulatory system, and might increase the risk of schizophrenia, autism, and mental disorders (Smith et al., 2007). Nevertheless, there are a limited number of studies addressing this relationship, and it is difficult to measure the attributable risk, if any, from multiple causes, mainly those with low effect to complex conditions. Moreover, it is complicated to separate if the cause of negative outcomes was the causative agent or the body response to the infection. For example, fever has been associated with several birth defects, such as neural tube defects, congenital heart defects, and oral clefts (Dreier et al., 2014).

With regard to the risk of infecting directly the embryo/fetus and disrupting its development, SARS-CoV-2 has been shown to affect several organs in human adults, which increases the probability of different developing organs being infected and affected in embryos or fetuses, if transplacental passage occurs. To reach the developing baby, the virus would need to bind to appropriated receptors. SARS-CoV-2 enters the cell through the binding of viral spike (S) protein to the cellular receptor Angiotensin-converting Enzyme 2 (ACE2) and protein priming mediated by serine protease Transmembrane Protease Serine 2 (TMPRSS2), which cleaves the S protein (Hoffmann et al., 2020), both also used by SARS-CoV (Li et al., 2003; Glowacka et al., 2011; Iwata-Yoshikawa et al., 2019). The *ACE2* gene is expressed in endometrium and different cell types in early placenta, including stromal cells and syncytiotrophoblast (Wang et al., 2019; Li M *et al*., 2020; Papatheodorou et al., 2020). Interestingly, in early pregnancy, *ACE2* has a very low level of expression in extravilous trophoblast and such expression increases as the pregnancy carries on (Li M *et al*., 2020). In the developing embryo, *ACE2* is abundantly expressed in many different organs including kidney, heart and liver (Cardoso-Moreira et al., 2020; Papatheodorou *et al*., 2020). Interestingly, MERS-CoV receptor, DPP4 is also highly expressed in placenta and endometrium (Wang *et al*., 2019; Papatheodorou *et al*., 2020), and ubiquitously expressed in many tissues of the embryo/fetus including liver, brain, reproductive organs, heart and kidney liver (Cardoso-Moreira *et al*., 2020; Papatheodorou *et al*., 2020). This indicates that the virus could reach the embryo, infecting it and leading to more severe adverse outcomes, as for MERS that cause, among others, intrauterine growth restriction (Di Mascio et al., 2020; Schwartz, 2020). Therefore, changes in ACE2 availability could affect directly the developing embryo.

Concerning the *TMPRSS2* gene, it was found to be expressed in endometrium and placenta, but with lower levels than *ACE2* (Wang et al., 2019; Papatheodorou et al., 2020). As for *ACE2* expression, *TMPRSS2* is modestly expressed early pregnancy and increases over time (Li M et al., 2020). Although presenting a moderate and more limited expression, *TMPRSS2* is also co-expressed with *ACE2* in the embryonic heart and lungs, suggesting a possible embryonic/fetal infection during pregnancy would be possible (Cardoso-Moreira et al., 2020; Li M *et al*., 2020; Papatheodorou *et al*., 2020). Considering that both *ACE2* and *TMPRSS2* are co-expressed in important placental cells and such expression increases during pregnancy (Li M *et al*., 2020), one could consider that in later pregnancy these tissues would be more susceptible for viral effects and adverse outcomes as observed for SARS and MERS could also be seem in pregnancies affected by COVID-19.

Another point that must be considered for risks assessment of COVID-19 and repercussion for mother and baby, is about SARS-Cov-2 virulence and its mutation rate. Although SARS-CoV-2 seems to be less harmful to pregnancy than SARS-Cov and MERS-Cov, we should be aware that the viruses can accumulate stochastic mutations over time, especially when it spreads quickly over the globe as SARS-CoV-2. So far, evidences have shown that the new coronavirus has a lower mutation rate (or mutates slowly) when compared to other viruses, including SARS-CoV and Influenza (Cyranoski, 2020; Yong *et al*., 2020). Nevertheless, it is important to keep in mind that a single mutation in tge Zika virus genome was enough to increase the viral neurotropism (Yuan et al., 2017). Such mutation occurred before the 2013 outbreak in French Polynesia and enhanced ZIKV virulence, leading to the increased incidence of microcephaly in Brazil (Yuan *et al*., 2017). This shows that depending on the mutation, it should be enough to increase the odds of the virus being harmful to the developing baby. In addition, ZIKV teratogenesis seems to affect different populations with different severity, as it was observed in the Brazilian Northeast region, which presented more cases of microcephaly than othes. These findings could indicate that the potential of SARS-CoV-2 being a teratogenic agent would depend on different factors, including the host genetic background and genetic variability of the pathogen. Indeed, some studies have shown that genetic background is important not only for infection susceptibility but also to pregnancy outcomes (Caires-Júnior *et al*., 2018). Finally, teratogenic effects of an agent do not necessarily need to be as severe and evident as the most popular such as ZIKV or thalidomide. Therefore, early pregnancy loss, less severe growth restrictions, respiratory tract defects or preterm labor might not be easily observed at the first moment.

In summary, taking into account that it is a new infection caused by a new agent that has been described less than one year ago, we cannot rule out that SARS-CoV-2 could cause adverse pregnancy outcomes. In order to better understand the relationship between COVID-19 and pregnancy, it is fundamental to elucidate if and how the virus disrupts embryo development from different approaches (Figure 1). In this sense, experimental studies in animals, and observational studies in humans evaluating host and pathogen genetics could give insights on its teratogenic potential, as well aas viral molecular mechanisms of action. Clearly, more research in the the epidemiological profile of mothers affected by COVID-19 must be performed, especially studies that allow to detect less evident congenital anomalies at birth. Surveillance and follow-up on miscarriage rate or children being born with low weight, immunological conditions, respiratory diseases and even minor congenital anomalies should be done. A long-term follow-up of babies exposed to SARS-CoV-2 should be performed in order to identify if the virus has caused any functional anomaly. This type of surveillance is especially important in those cases in which a cytokine storm was observed; this phenomenon is characterized by an elevated production of proinflammatory cytokines/chemokines, which contributes to acute lung injury (Liu H et al., 2020). Regarding maternal complications, several measures have been proposed by Rasmussen et al. (2020). In some cases, simple approaches as fetal heart rate can be used as an indicator of clinical condition, since changes in heart rate are related to the worsening of the respiratory condition, and some pregnant women with SARS and MERS did develop respiratory failure (Rasmussen *et al*., 2020). Considering the recommendations aforementioned, it is essential to perform a multidisciplinary approach focused on both mother and baby, including a risk evaluation in a Teratogen Information Service. In Brazil, our Teratogen Information Service (TIS) at the Medical Genetics Service in Hospital de Clinicas de Porto Alegre (University Hospital) has worked on these risk assessments since 1990. The Brazilian TIS, called SIAT (Sistema de Informação sobre Agentes Teratogênicos, in Portuguese), is a free telephone/email service that provides information on reproductive risks related to the exposure of pregnant women to chemical, physical and biological agents. In addition to giving updated information to clinics (clinicians), SIAT also acts in the investigation of the teratogenicity of environmental agents by following and observing the results of pregnancies, including those exposed to viral infection, such as Rubella (Minussi et al., 2008), H1N1 (Silva et al., 2012) and Zika virus (Schuler-Faccini et al., 2016). Finally, from different approaches and collaborative initiatives, such as COVI-Preg Registry (Panchaud et al., 2020), health care workers who are taking care of pregnant women affected by COVID-19 as well as scientists and surveillance systems on birth defects must be watchful in order to investigate, detect and prevent possible embryonic damaging effects of the new coronavirus.

**Figure 1 -:**
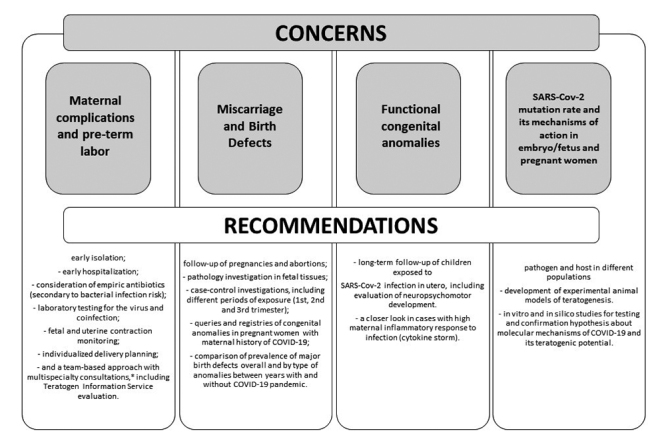
Summary of concerns (grey squares) and recommendations (white boxes) about COVID-19 infection during pregnancy.
